# Benzoic acid (inhalable fraction): MAK Value Documentation, addendum – Translation of the German version from 2023

**DOI:** 10.34865/mb6585e10_4ad

**Published:** 2025-12-22

**Authors:** Andrea Hartwig, MAK Commission

**Affiliations:** 1 Institute of Applied Biosciences. Department of Food Chemistry and Toxicology. Karlsruhe Institute of Technology (KIT) Adenauerring 20a, Building 50.41 76131 Karlsruhe Germany; 2 Permanent Senate Commission for the Investigation of Health Hazards of Chemical Compounds in the Work Area. Deutsche Forschungsgemeinschaft, Kennedyallee 40, 53175 Bonn, Germany. Further information: Permanent Senate Commission for the Investigation of Health Hazards of Chemical Compounds in the Work Area | DFG

**Keywords:** benzoic acid, irritation, MAK value, maximum workplace concentration, inhalable fraction, developmental toxicity, skin absorption, Luft, air

## Abstract

The German Senate Commission for the Investigation of Health Hazards of Chemical Compounds in the Work Area (MAK Commission) has re-evaluated the data for benzoic acid [65-85-0] to derive an occupational exposure limit value (maximum concentration at the workplace, MAK value) for the inhalable fraction. The critical effects are severe irritation in the eyes and lung toxicity. For this reason, irritation is assumed to occur also in the upper respiratory tract. Inhalation studies investigating the inhalable fraction of benzoic acid in humans or animals are not available. Therefore, data for similarly strong acids are used for the evaluation of the local effects on the respiratory tract. In analogy to the MAK value derived for phosphoric acid, a MAK value of 2 mg/m^3^ I (inhalable fraction) has been established. This represents the “worst case” for benzoic acid due to its weaker acidity. Peak Limitation Category I with an excursion factor of 2 has been set in analogy to the classification made for phosphoric acid. There are no valid prenatal developmental studies of benzoic acid available. Benzoates are classified in Pregnancy Risk Group C at the MAK value of 10 mg/m^3^. As the benzoate anion is responsible for the systemic effects of benzoic acid and the acid has a lower MAK value, Pregnancy Risk Group C is valid also for the inhalable fraction of benzoic acid at the MAK value of 2 mg/m^3^. Dermal uptake is expected to contribute to systemic toxicity and benzoic acid remains designated with “H” (for substances which can be absorbed through the skin in toxicologically relevant amounts).

**Table:** 

**MAK value (2022)**	**0.39 ml/m^3^ (ppm) ≙ 2 mg/m^3^ I (inhalable fraction)**
**Peak limitation (2022)**	**Category I, excursion factor 2**
	
**Absorption through the skin (2022)**	**H**
**Sensitization**	**–**
**Carcinogenicity**	**–**
**Prenatal toxicity (2022)**	**Pregnancy Risk Group C**
**Germ cell mutagenicity**	**–**
	
**BAT value**	**–**
	
Chemical name (IUPAC)	benzoic acid
CAS number	65-85-0
Structural formula	Structural formula of benzoic acid. 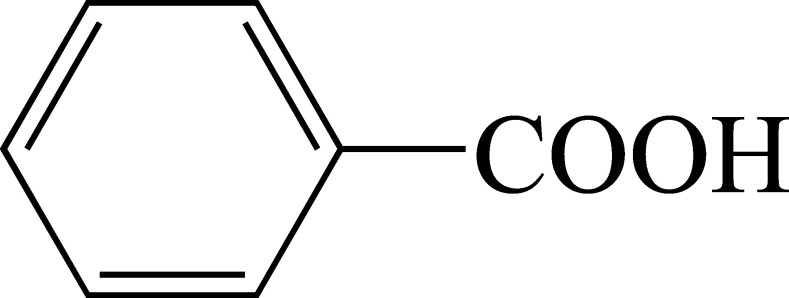
Molecular formula	C_7_H_6_O_2_
Molar mass	122.12 g/mol
Vapour pressure at 25 °C	0.0009 hPa (Hartwig and MAK Commission 2018 b)
pKa value	4.19 (Hartwig and MAK Commission 2018 b)
**1 ml/m^3^ (ppm) ≙ 5.067 mg/m^3^**	**1 mg/m^3^ ≙ 0.197 ml/m^3^ (ppm)**

Note: The substance can occur simultaneously as vapour and aerosol. It triggers pseudoallergic reactions (see Greim 1995, available in German only).

For benzoic acid, documentation from 1986 (Henschler 1986, available in German only) and an addendum from 1995 (Greim 1995) describing allergenic effects are available. In another addendum from 2017 (Hartwig and MAK Commission 2018 b) also the alkali benzoates were included, since in aqueous solution the benzoates are in equilibrium with the benzoic acid depending on the pH value.

For benzoic acid, the critical effect is lung toxicity. As irritation to the upper respiratory tract is also expected, a MAK value for the I (inhalable) fraction of benzoic acid is derived in this addendum. The R (respirable) fraction of benzoic acid and the alkali benzoates are not the subject of this addendum. The derivation of the MAK value for the R fraction of benzoic acid can be found in the addendum from 2017 (Hartwig and MAK Commission 2018 b).

## Manifesto (MAK value/classification)

The critical effects of benzoic acid are severe irritation in the eyes and lung toxicity after inhalation exposure.

**MAK value. **Data suitable for deriving a MAK value in humans are not available. Inhalation studies investigating the I fraction of benzoic acid in animals are likewise not available. Therefore, data for similarly strong acids are used for the evaluation of the local effects on the respiratory tract. The acid strength of benzoic acid, with a pKa value of 4.19, is in the range of the strength of succinic acid (pKa values 4.21 and 5.64), adipic acid (pKa values 4.34 and 5.44) and the somewhat more acidic tartaric acid (pKa values 2.98 and 4.34). The pKa value of phosphoric acid is 2.2 (most acidic proton) (Greim 2006, available in German only).

A MAK value for the I fraction of benzoic acid can be established by analogy with phosphoric acid (Greim 2006), as already been done for tartaric acid (Hartwig 2015, available in German only), succinic acid (Hartwig and MAK Commission 2018 c) and adipic acid (Hartwig and MAK Commission 2018 a). Phosphoric acid has a MAK value of 2 mg/m^3^ I, derived from a NOAEC (no observed adverse effect concentration) of 37.5 mg phosphoric acid/m^3^ from a 13-week inhalation study in rats (Greim 2006).

By analogy with phosphoric acid (MAK value 2 mg phosphoric acid/m^3^ ≙ 0.02 mmol/m^3^ ≙ 2.44 mg benzoic acid/m^3^), a MAK value of 2 mg/m^3^ I has been established for benzoic acid until suitable data become available. This represents the worst case for benzoic acid due to its weaker acidity. A systemic effect of benzoic acid is to be expected only at much higher concentrations (see Hartwig and MAK Commission 2018 b).

**Peak limitation. **Because of the critical local effect, the I fraction of benzoic acid has been assigned to Peak Limitation Category I. As the MAK value of benzoic acid has been established by analogy with that for phosphoric acid, the same excursion factor of 2 as for phosphoric acid (Greim 2006) has been set for peak limitation for benzoic acid.

**Prenatal toxicity. **Three studies are available for benzoic acid, all of which were not conducted according to valid test guidelines and have considerable shortcomings. However, the systemic effects of benzoic acid are likely to be similar to those of the salt, as these effects are mediated via the benzoate formed in the organism. In a prenatal developmental toxicity study in Wistar rats, sodium benzoate led to foetotoxic effects such as a decreased number of live foetuses, decreased foetal weights, delayed ossification and increased incidences of skeletal, external and internal variations and malformations at about 1850 mg/kg body weight and day with concomitant maternal toxicity. The NOAEL (no observed adverse effect level) for developmental and maternal toxicity was approximately 1340 mg sodium benzoate/kg body weight and day. In prenatal developmental toxicity studies in mice, rabbits and hamsters, no developmental or maternal toxicity occurred up to the highest doses tested of 175 mg, 250 mg and 300 mg sodium benzoate/kg body weight, respectively. As the margins between the concentrations in air calculated by toxicokinetic extrapolation and the MAK value are sufficiently large, the benzoates were assigned to Pregnancy Risk Group C with a MAK value of 10 mg/m^3^ I (Hartwig and MAK Commission 2018 b). Benzoic acid has a lower MAK value than the benzoates of 2 mg/m^3^ I. The margin between this MAK value and the NOAEL for developmental toxicity of the benzoate is thus 5 times as large. Due to the larger margin, Pregnancy Risk Group C is valid also for the I fraction of benzoic acid at a MAK value of 2 mg/m^3^ I.

**Absorption through the skin. **There are no new data available.

The possible contribution of dermal absorption to systemic toxicity is not negligible; benzoic acid therefore remains designated with an “H” (for substances which can be absorbed through the skin in toxicologically relevant amounts) (Hartwig and MAK Commission 2018 b).
